# Alteration of JNK-1 Signaling in Skeletal Muscle Fails to Affect Glucose Homeostasis and Obesity-Associated Insulin Resistance in Mice

**DOI:** 10.1371/journal.pone.0054247

**Published:** 2013-01-17

**Authors:** Martin Pal, Claudia M. Wunderlich, Gabriele Spohn, Hella S. Brönneke, Marc Schmidt-Supprian, F. Thomas Wunderlich

**Affiliations:** 1 Max Planck Institute for Neurological Research, Institute for Genetics, University of Cologne and Cologne Excellence Cluster on Cellular Stress Responses in Aging-Associated Diseases (CECAD) and Center of Molecular Medicine Cologne (CMMC), Cologne, Germany; 2 Mouse Phenotyping Core Facility of Cologne Excellence Cluster on Cellular Stress Responses in Aging-Associated Diseases (CECAD), Cologne, Germany; 3 Molecular Immunology and Signaltransduction, Max Planck Institute for Biochemistry, Munich, Germany; Warren Alpert Medical School of Brown University, United States of America

## Abstract

Obesity and associated metabolic disturbances, such as increased circulating fatty acids cause prolonged low grade activation of inflammatory signaling pathways in liver, skeletal muscle, adipose tissue and even in the CNS. Activation of inflammatory pathways in turn impairs insulin signaling, ultimately leading to obesity-associated type 2 diabetes mellitus. Conventional JNK-1 knock out mice are protected from high fat diet-induced insulin resistance, characterizing JNK-1-inhibition as a potential approach to improve glucose metabolism in obese patients. However, the cell type-specific role of elevated JNK-1 signaling as present during the course of obesity has not been fully elucidated yet. To investigate the functional contribution of altered JNK-1 activation in skeletal muscle, we have generated a ROSA26 insertion mouse strain allowing for Cre-activatable expression of a JNK-1 constitutive active construct (JNK^C^). To examine the consequence of skeletal muscle-restricted JNK-1 overactivation in the development of insulin resistance and glucose metabolism, JNK^C^ mice were crossed to Mck-Cre mice yielding JNK^SM-C^ mice. However, despite increased muscle-specific JNK activation, energy homeostasis and glucose metabolism in JNK^SM-C^ mice remained largely unaltered compared to controls. In line with these findings, obese mice with skeletal muscle specific disruption of JNK-1, did not affect energy and glucose homeostasis. These experiments indicate that JNK-1 activation in skeletal muscle does not account for the major effects on diet-induced, JNK-1-mediated deterioration of insulin action and points towards a so far underappreciated role of JNK-1 in other tissues than skeletal muscle during the development of obesity-associated insulin resistance.

## Introduction

The increasing prevalence of obesity in the population of developed countries represents a serious health problem, since obesity is a major risk factor for the development of insulin resistance, hyperglycemia, and metabolic syndrome. It is therefore important to understand the molecular mechanism that accounts for obesity-induced insulin resistance. Weight gain during the course of obesity induces low grade chronic inflammation in white adipose tissue (WAT) and liver leading to the release of pro-inflammatory cytokines such as TNF-α into circulation [1]. TNF-α inhibits insulin action in classical insulin target tissues such as skeletal muscle, liver and adipose cells, thereby resulting in impaired glucose homeostasis [2], [3]. Here, TNF-stimulated activation of the cJun N-terminal kinase (JNK) and inhibitor of κB kinase (IKK) signaling pathways inhibit insulin action in a tissue-specific manner [2], [4]–[6]. Moreover, JNK activation mediates insulin resistance by other activators such as free fatty acids and ER stress, both of which are elevated during the course of obesity [7], [8]. JNK activation leads to inhibitory serine phosphorylation of insulin receptor substrate (IRS) proteins inhibiting insulin signaling thus causing insulin resistance and ultimately contributing to the development of type 2 diabetes mellitus [9].

JNKs are expressed by three different genes, JNK-1, -2 and -3, all of which generate various JNK-isoforms by alternative splicing mechanisms [10]. JNK-1 and -2 are ubiquitously expressed, while JNK-3 expression is restricted mainly to brain and heart [11]. Recent studies identified JNK-1 as the major isoform contributing to the development of obesity-associated insulin resistance, since mouse mutants deficient for JNK-1 but not those for JNK-2 are largely protected from the development of obesity and obesity-associated insulin resistance in both diet- and genetically-induced obesity models [12], [13]. However, the tissue-specific contribution of JNK-1-deficiency to the development of obesity-associated insulin resistance *in vivo* is poorly understood. While adipose-tissue-specific JNK-1-deficiency confers some protection to obesity-associated insulin resistance [14], analyses of the contribution of JNK-1-action in the hematopoetic system using bone marrow transplantation have yielded conflicting results [15], [16]. On the other hand, myeloid lineage-cell-specific disruption of JNK-1 failed to protect from obesity-associated insulin resistance, ruling out a major contribution of macrophages to this phenomenon [14]. More recently, skeletal muscle-specific JNK-1 disruption reported by Sabio and colleagues revealed a potential role of JNK-1 signaling in the control of systemic insulin sensitivity in high fat diet-induced obesity [17]. Specifically, muscle-specific JNK-1-deficiency left body weight gain and glucose tolerance unaltered during the course of obesity, but ameliorated insulin sensitivity and insulin-stimulated AKT phosphorylation in liver and WAT but not in the JNK-1-deficient muscle [17]. Interestingly, another study using disruption of JNK-1 in hepatocytes demonstrated that hepatic JNK-1-signaling in lean mice is required to prevent the development of steatosis, while in diet-induced obese mice, JNK-1-deficiency was unable to further aggravate lipid accumulation in hepatocytes [18]. The most promising candidate site in which disruption of JNK-1 could largely reflect the lean phenotype of JNK-1 knock out mice was recently identified as the central nervous system. We and others could show, that neuronal and pituitary JNK-1 deficiency has significant impact on insulin sensitivity and the growth hormone/insulin like growth factor axis [19], [20]. Mice carrying a central JNK-1 inactivation were small sized and had reduced body weight accompanied with increased insulin sensitivity compared to controls [19].

However, none of these mouse mutants completely phenocopied the effect observed in conventional JNK-1 knock out mice indicating that other cell types or combinations thereof are involved in mediating JNK-1′s effect on insulin sensitivity. Despite the current cell type-specific knowledge about JNK-1 signaling in the development of obesity-induced insulin resistance, mimicry of JNK-1 overactivation as present in the course of obesity has not been elucidated yet. Here, we investigated mice with isolated transgenic overactivation of JNK-1 in skeletal muscle for alterations in glucose metabolism and energy homeostasis. However, besides cell type-specific aggravation of JNK-1 signaling in these organs, the impact on insulin sensitivity and glucose homeostasis was minor. To further investigate the cell type specific function of JNK-1 in obesity, we have generated conditional JNK-1 knock out mice in skeletal muscle. Strikingly, when exposed to high fat diet, obesity developed to a similar extent in JNK-1 mutants and controls and obesity induced a similar inhibition of insulin action in controls and mutants despite successful reduction of JNK-1 expression in these tissues. Taken together our data reveal, that JNK-1 action in skeletal muscle fails to affect the development of obesity and obesity-associated insulin resistance.

## Materials and Methods

### Animal Care

Care of all animals was within institutional animal care committee guidelines. All animal procedures were conducted in compliance with protocols and approved by local government authorities (Bezirksregierung Köln, Cologne, Germany). Animals were fed either normal chow diet (NCD) (2918 Teklad Global 18% Protein Rodent Diet, Teklad Diets, Madison, Wi., USA) containing 53.5% carbohydrates, 18.5% protein, and 5.5% fat (12% of calories from fat), or a high-fat diet (HFD) (C1057; Altromin, Lage, Germany) containing 32.7% carbohydrates, 20% protein, and 35.5% fat (55.2% of calories from fat) starting from 3 weeks of age. All animals had access to water and food ad libitum. Body weight was measured once per week. Food intake was quantified daily over a period of 7 days. Mice were sacrificed using CO_2_.

### Generation of JNK^SM-C^ Mice

The JNK^C^ construct consisting of a JNKK-2D(MKK-7D)-JNK-1 fusion protein was created by conventional cloning techniques. Sequences encoding the JNKK2(MKK7)-JNK1 fusion protein [36] or a constitutively active MKK7D [37] were subcloned into pBluescript flanked by AscI sites. The fragment containing the S271D and T275D mutations was then excised by NotI-BsmBI from the MKK7D containing plasmid and cloned NotI-BsmBI into the JNKK2(MKK7)-JNK1 containing plasmid, yielding a MKK7D-JNK1 containing plasmid. The MKK7D-JNK1 (JNK^C^) sequences were then inserted into the AscI site of the STOP-EGFP-ROSA targeting vector that additionally contained a CAG promoter. Sequences are available upon request. 10^7^ Bruce4 ES cells were transfected with the targeting vector and positive clones were identified using the ROSA probe [38] in Southern Blot analysis. Positive ES cell clones were used to generate the ROSA JNK^C^ mouse strain, which was further crossed to Mck-Cre mice to generate JNK^SM-C^ mice. JNK^SM-C^ mice were genotyped for the presence of the JNK^C^ construct by performing PCR analysis using primers 5_MKK7_JNK1CA (5′-AAG-AAC-TGC-AAG-ACG-GAC-TT-3′) and 3_MKK7_JNK1CA (5′- TTC-ATA-AGA-ACT-AGC-TCT-CTG-T-3′).

### Generation of JNK-1^SM-KO^ and JNK-1^KO^ Mice

To generate JNK-1^SM-KO^ male mice Mck-Cre mice [21] were crossed to JNK-1^FL/FL^ mice [19]. Further, JNK-1^FL/FL^ mice were crossed to deleter Cre mice to yield JNK-1^KO^ mice. In all experiments, littermates carrying the loxP-flanked JNK-1 allele were used as controls. Genotyping for the Mck-Cre trangene was performed by PCR by using primers mck5 (5′-GTT-CTT-AAG-TCT-GAA-CCC-GG-3′), mck3 (5′-GTC-TGG-ATG-ACA-TCG-TCC-AG-3′) and Cre_intern_rev3 (5′-ATG-TTT-AGC-TGG-CCC-AAA-TGT-3′). For identifying the JNK-1^FL/FL^ and the JNK-1^KO^ alleles, the primers 5J1loxNT (5′-ACA-TGT-ACC-ATG-TAC-TGA-CCT-AAG-3′), 3J1loxNT (5′-CAT-TAC-TCT-ACT-CAC-TAT-AGT-AAC-3′) and 3J1deltaNT (5′-GAT-ATC-AGT-ATA-TGT-CCT-TAT-AG-3′) were used.

### Analysis of Body Composition

Lean mass and body fat content of live animals was determined using the NMR Analyser Minispec (Bruker Optik).

### Indirect Calorimetry

Indirect calorimetry was measured in a PhenoMaster System (TSE Systems) as previously described [22], [23]. After an overnight acclimatization, parameters of indirect calorimetry were measured for 48 to 72 h.

### Glucose and Insulin Tolerance Test

Glucose-tolerance tests were performed on animals fasted for 16 h. Animals were injected with either 2 g/kg body weight of glucose or 0.75 units/kg body weight of insulin (Insuman Rapid, Sanofi Aventis) into the peritoneal cavity. Blood glucose concentrations were determined by using an automated glucose monitor (GlucoMen) for the indicated time points.

### Insulin and Leptin Determination from Serum

Plasma insulin and leptin concentrations were examined by ELISA (mouse/rat insulin ELISA, #INSKR020, mouse leptin ELISA, #900-019, Chrystal Chem, BD Bioscience PharMingen) according to the manufacturer's protocol.

### Southern Blot Analysis

For Southern blot, 15 µg of DNA isolated from embryonic stem cells was digested over night with 100 U of EcoRI. The DNA fragments were separated by agarose gel electrophoresis and transferred onto Hybond-XL™- membranes (Amersham) by alkaline capillary transfer. 80 ng DNA of the ROSA26 probe was radioactively labeled with 2.5 mCi^[α32P]^dATP (Amersham) using the Ladderman™ Labeling Kit (Takara) as previously described [24].

### Gene Expression Analysis

Quantitative expression for JNK^C^ mRNA (Hs01548508_m1, Applied Biosystems, Foster City, USA) was determined by using whole-muscle RNA isolated by TRIzol (Invitrogen). RNA was reversely transcribed with High Capacity cDNA Reverse Transcription Kit (Applied Biosystems) and amplified by using TaqMan Gene Expression Master Mix (Applied Biosystems). Relative expression of mRNAs was determined by using standard curves based on cDNA derived from the respective tissues, and samples were adjusted for total RNA content by Hprt1 (Mm00446968_m1, Applied Biosystems), Gusb (Mm00446953_m1, Applied Biosystems) and Tfrc (Mm00441941_m1, Applied Biosystems) quantitative PCR. Calculations were performed by a comparative cycle threshold (Ct) method: starting copy number of test samples was determined in comparison with the known copy number of the calibrator sample (ddCt). The relative gene copy number was calculated as 2^-ddCt^. Quantitative PCR was performed on an ABI PRISM 7900 Sequence Detector (Applied Bioscience). Assays were linear >4 orders of magnitudes.

### Insulin Signaling

Mice were anesthetized by intraperitoneal injection of ketamin (100 µg/µl, Albrecht GmbH, Germany). 5 mU of human regular insulin (Insuman Rapid, Sanofi Aventis) were injected into the inferior *vena cava*. Skeletal muscle and liver were collected 2, 5 and 7 min after injection and processed as previously described [21].

### Western Blot Analysis

Muscle and liver tissues were homogenized with a polytron homogenizer (IKA Werke) in protein lysis buffer and centrifuged at 13,000 g for 1 h at 4°C. Proteins in the supernatant were electrophoresed by SDS–polyacrylamide gel electrophoresis (10%) and transferred to PVDF membranes (Bio-Rad). Membranes were probed with the following antibodies: JNK 1/3 (Santa Cruz Biotechnology Inc.), phospho-JNK (#4668, Cell Signaling Technology Inc.), AKT (pan) (#4685, Cell Signaling Technology Inc.), phospho-AKT (#9271, Cell Signaling Technology Inc.), Calnexin (#20880, Calbiochem, Germany) and goat anti-rabbit conjugated to HRP (A6154, Sigma-Aldrich).

### JNK Activity Assays

JNK activity assays were performed as previously described [25]. Human cJun peptide (residues 1–79) fused to glutathione S-transferase (Biovision, USA) was bound to glutathione sepharose beads 4B (GE Healthcare, Germany) overnight. After 2 washing steps, skeletal muscle lysates from WT and JNK^SM-C^ mice were incubated overnight with the GST-c-Jun fusion beads. Kinase reaction was induced by adding 200 µM [γ-^32^ P]-ATP (3000 Ci/mmol) (Hartmann Analytic, Germany). After SDS-PAGE, phosphorylation of c-Jun was detected by using a Typhoon Trio Imager (GE Healthcare, Germany).

### Statistical Analyses

Datasets were analyzed for statistical significance by using a two-tailed, unpaired Student's *t* test and one-or two-way analysis of variance (ANOVA) (PASW Statistics 18.0). *P* values <0.05 were considered significant. For visual clarity, instead of standard deviations, means +/− standard errors of the mean (SEM) are depicted in all figures.

## Results

### JNK is Activated in Skeletal Muscle of Obese Mouse Models

**Figure 1:**
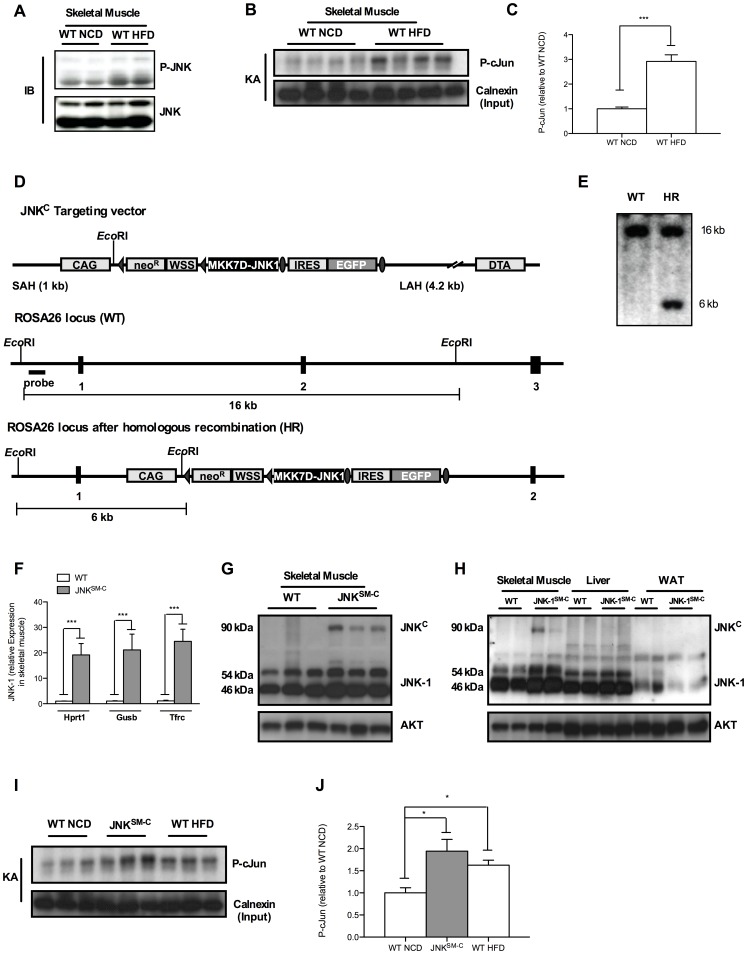
Muscle specific JNK overactivation. (A) Western Blot analysis (IB) of protein lysates isolated from muscles of WT mice at 20 weeks of age when exposed to NCD and HFD using P-JNK and JNK antibodies. (B) JNK-kinase assay of protein lysates isolated from muscles of WT mice at 20 weeks of age when exposed to NCD and HFD. Calnexin antibodies were used for input control. (C) Quantitation of radioactively labeled cJun peptide in skeletal muscle of NCD fed WT and HFD WT mice. (D) Targeting strategy for the Cre-activatable JNK^C^ construct into the ubiquitously expressed ROSA26 locus. The CAG modified STOP-EGFP-ROSA targeting vector was described elsewhere. In the unique AscI site, a fusion cDNA between the mutant MKK7D [36] and JNK-1 [37] was inserted to generate the final ROSA JNK^C^ targeting vector. Homologous recombination between the homology arms and the genomic ROSA26 locus generated the targeted allele which was identified by Southern Blot analysis of EcoRI-digested clonal DNA using the ROSA probe [38] resulting in the 16 kb WT band besides the 6 kb targeted band as shown in (E). Such positive C57/BL6-derived ES cell clones were used to generate the ROSA JNK^C^ mouse strain. (F) Determination of relative JNK-1 mRNA expression in skeletal muscle of WT and JNK^SM-C^ mice by quantitative realtime PCR using Hprt1, Gusb and Tfrc as housekeeping genes (n = 8). (G) Western Blot analysis of protein lysates isolated from skeletal muscle of WT and JNK^SM-C^ mice using JNK-1 and AKT antibodies. (H) Expression of the JNK^C^ fusion protein only in skeletal muscle of JNK^SM-C^ mice indicated by Western blot analysis. (I) Functionality of the JNK^C^ construct in skeletal muscle of JNK^SM-C^ mice was revealed by increased phosphorylation of cJun in JNK kinase assay (KA) experiments. (J) Quantitation of radioactively labeled cJun peptide in skeletal muscle of NCD fed WT, JNK^SM-C^ and HFD WT mice. Values are means ± SEM. ***, p≤0.001.

Obesity-induced JNK activation has been described to result in the development of insulin resistance in various organs by inhibitory serine phosphorylation of IRS proteins [12]. In detail, inflammatory cytokines that are elevated during the course of obesity such as TNF-α activate upstream kinases of JNK that phosphorylate JNK-1 and JNK-2 at specific Thr and Tyr residues resulting in JNK activation that can be monitored by several means. To address whether JNK is activated in response to obesity in skeletal muscle, we examined phosphorylation of JNK by using phospho-specific antibodies. Consistent with previous reports, JNK phosphorylation was increased in the muscle of mice that were exposed to high fat diet (HFD) feeding for 14 weeks when compared to organs of mice receiving normal chow diet (NCD) (Fig. 1A). Moreover, to quantify this increase in JNK activity and to prove that the elevated JNK activation under HFD conditions translates also into further downstream signaling, we elucidated JNK activity by a kinase assay using GST coupled cJun peptide as a substrate in lysates obtained from skeletal muscle of NCD- and HFD-fed mice (Fig. 1B). This analysis revealed a significant increase of JNK activity in skeletal muscle when mice were exposed to HFD feeding as revealed by quantitation of radioactively labeled cJun peptide (Fig. 1C). Thus, our data reveal a 2.8-fold increased JNK activation in skeletal muscle under obese conditions when compared to lean mice.

### Successful Transgenic Mimicking of Diet-induced JNK-activation in Skeletal Muscle

Obesity-induced JNK activation has been closely linked to result in the development of insulin resistance. To mimic such JNK overactivation, we employed a novel mouse model allowing for Cre-activatable cell type-specific overactivation of JNK signaling. To this end, we generated mice carrying a constitutive active version of JNK (JNK^C^) in the ROSA26 locus (ROSA JNK^C^), by targeted insertion of a MKK-7D-JNK-1 fusion-gene, whose expression is prevented by a loxP-flanked transcriptional stop cassette (Fig. 1D). Upon Cre-mediated recombination, the stop cassette is excised, thus permitting the compound expression of the JNK^C^ construct and EGFP. The correct integration of the JNK^C^ transgene into the ROSA26 locus of C57BL6-derived Bruce-4 embryonic stem cells was confirmed by Southern blot analysis using the external ROSA26 probe (Fig. 1E). Correctly targeted ES cell clones were used to generate the conditional ROSA JNK^C^ mouse strain on a pure C57/BL6 background. By crossing the ROSA JNK^C^ to Mck-Cre mice, we created a mouse model with isolated skeletal muscle-specific overactivation of JNK signaling (JNK^SM-C^). Indeed, assessment of JNK expression in skeletal muscle revealed not only successful overexpression of the JNK^C^ construct on the RNA level (Fig. 1F), but also on the protein level (Fig. 1G). Of note, to exclude transcriptional regulation of the housekeeping gene by JNK, we compared and normalized relative JNK expression to the expression of Hprt1, Gusb and Tfrc genes, respectively (Fig. 1F). Nevertheless, JNK^C^ expression was detected exclusively in skeletal muscle but not in liver and WAT of JNK^SM-C^ mice confirming the skeletal muscle specific excision of the loxP-flanked stop cassette by the Mck-Cre transgene (Fig. 1H). The presence of the JNK^C^ construct in these cells clearly over-activated JNK signaling compared to NCD-fed animals as revealed by increased phosphorylation of the downstream target cJun in a kinase assay of skeletal muscle lysates whereas the transgenic JNK activity reached similar activation as detected in skeletal muscle of HFD-fed animals (Fig. 1I, J). Importantly, to exclude variation of transgenic JNK^C^ overactivation in skeletal muscle of individual JNK ^SM-C^ mice, the densitometric quantitation of phosphorylated cJun and subsequent statistical analysis revealed similar distribution of P-cJun levels in this group (Fig. S1A, B and Fig. 1I, J). Taken together, we demonstrated that isolated JNK^C^ expression in skeletal muscle results in cell type-specific downstream JNK overactivation as present during the course of obesity.

### Isolated JNK Overactivation in Skeletal Muscle Fails to Affect Body Composition and Energy Homeostasis

**Figure 2:**
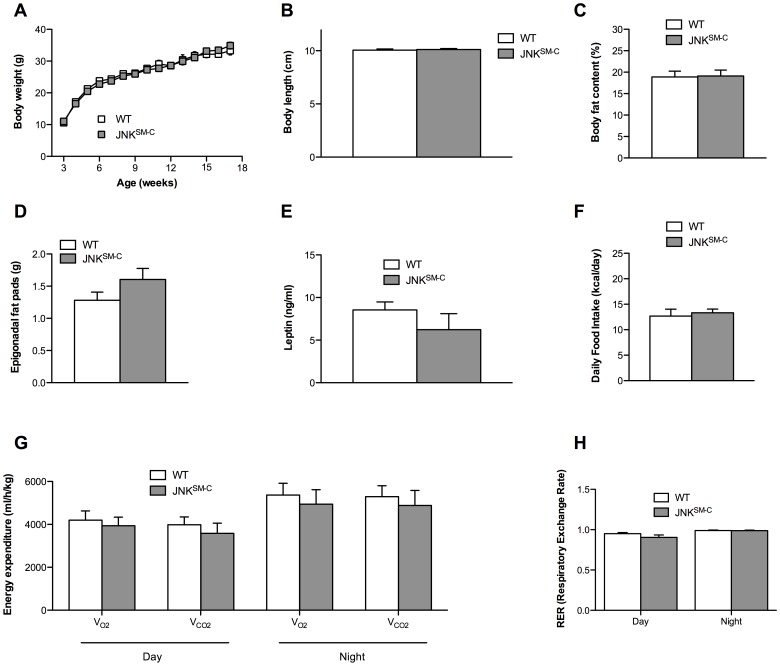
Unaltered body composition and energy homeostasis in JNK^SM-C^ mice. (A) The average bodyweight of control (open squares) mice was compared with JNK^SM-C^ (grey squares) mice from 3 to 17 weeks of age when feeding a NCD (n = 15). (B) Body length of control (white bar) and JNK^SM-C^ (grey bar) mice upon NCD feeding (n = 9–12). (C) Body composition of control (white bar) and JNK^SM-C^ (grey bar) mice when exposed to NCD was determined by using a Brucker minispec in week 17 (n = 10–12). (D) Weight of epigonadal fat pads from NCD fed control (white bar) and JNK^SM-C^ (grey bar) mice in week 17 (n = 9). (E) Serum leptin levels of control (white bar) and JNK^SM-C^ (grey bar) mice at the age of 17 weeks (n = 9). (F) Daily food intake of control (white bar) and JNK^SM-C^ (grey bar) mice upon NCD feeding at the age of 14 weeks (n = 5). (G) Energy expenditure revealed by the daily and nightly volume of O_2_ consumption and CO_2_ release of control (white bar) and JNK^SM-C^ (grey bar) mice upon NCD feeding (n = 5). (H) Respiratory exchange rate (RER) of control (white bar) and JNK^SM-C^ (grey bar) mice upon NCD feeding (n = 5). Values are means ± SEM.

Next, we aimed to address whether JNK^C^ expression in skeletal muscle affects weight gain by comparing body weight of control and JNK^SM-C^ mice from 3 to 17 weeks of age. Body weight measurements in cohorts of control and JNK^SM-C^ mice revealed that skeletal muscle-specific JNK^C^ expression failed to affect body weight gain over this time frame when compared to controls (Fig. 2A). Moreover, body length was unaltered in the mutant mice compared to controls at the age of 17 weeks (Fig. 2B). The result of equal weighty and sized JNK^SM-C^ and control mice was also confirmed by a comparable abundance of body fat as revealed by nuclear resonance spectroscopy analysis (Fig. 2C). In line with these results, mass of epigonadal fat pads of JNK^SM-C^ mice were comparable to control fat pads (Fig. 2D), which in turn translated into equal circulating amounts of the adipose-derived hormone leptin in both cohorts of mice (Fig. 2E). Though body composition was unaltered between control and JNK^SM-C^ mice, we conducted indirect calorimetry experiments of control and JNK^SM-C^ mice to exclude any alteration in food ingestion or energy homeostasis. In line with unaltered body weight and fat mass, daily food intake (Fig. 2F), energy expenditure (Fig. 2G) and respective respiratory exchange ratio (RER) (Fig. 2H) was unchanged in the two cohorts of mice. Collectively, these results demonstrate that JNK overactivation in skeletal muscle fails to affect body composition and energy expenditure.

### Overactivation of JNK in Skeletal Muscle Fails to Impair Insulin Signaling and Glucose Homeostasis in vivo

**Figure 3:**
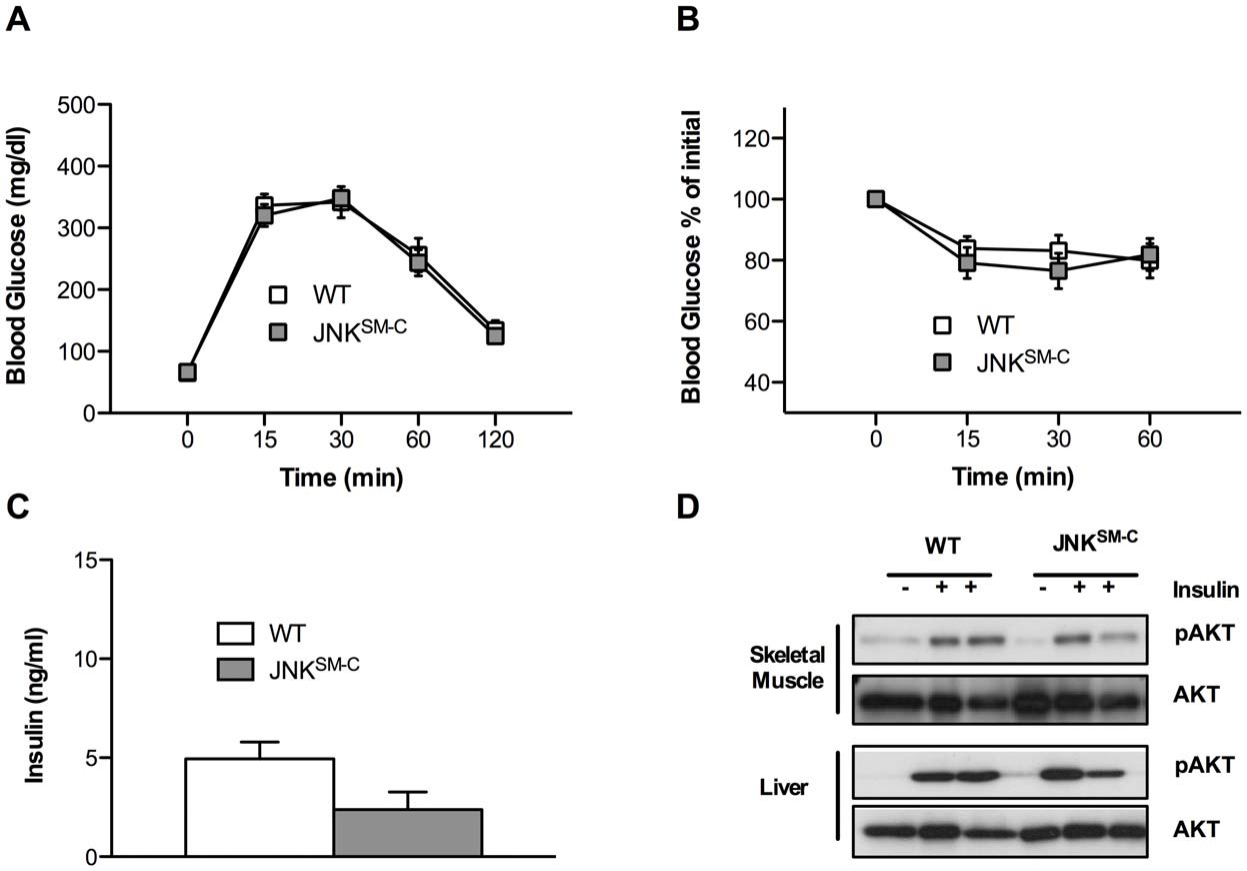
Unaltered glucose metabolism and insulin sensitivity in JNK^SM-C^ mice. (A) Glucose tolerance tests of control (white bar) and JNK^SM-C^ (grey bar) mice when feeding a NCD were performed at 11 weeks of age (n = 10–13). (B) Insulin tolerance tests of control (white bar) and JNK^SM-C^ (grey bar) mice upon NCD feeding were performed at 12 weeks of age (n = 13–15). (C) Insulin levels from sera isolated at week 17 from mice with the indicated genotypes upon NCD feeding determined by ELISA (n = 8–10). (D) Representative insulin-induced AKT phosphorylation of muscle and liver lysates isolated from control and JNK^SM-C^ mice when feeding NCD using Western Blot analysis with the indicated antibodies. Values are means ± SEM.

In light of the apparent lack of a major effect of skeletal muscle-specific JNK overactivation on body composition and energy expenditure, we wanted to address, whether isolated overactivation of JNK-dependent signaling in skeletal muscle (i.) causes overall alteration in whole body glucose homeostasis and (ii.) can impair insulin signaling in this organ *in vivo*. To this end, we performed glucose and insulin tolerance tests as well as examined circulating insulin concentrations and insulin-stimulated AKT phosphorylation as a measure of insulin sensitivity. Time-dependent glucose clearance from the blood upon intraperitoneal glucose challenge was identical between control and JNK^SM-C^ mice in glucose tolerance tests (Fig. 3A). Additionally, insulin sensitivity was unchanged in insulin tolerance tests in the two cohorts of mice (Fig. 3B). Moreover, circulating insulin concentrations were not affected by skeletal muscle specific JNK overactivation (Fig. 3C). Consistent with these observations, analysis of insulin-stimulated AKT-phosphorylation in skeletal muscle and liver of control and JNK^SM-C^ mice revealed that JNK overactivation in skeletal muscle did neither affect insulin-stimulated AKT-activation in muscle nor in liver (Fig. 3D). Taken together, these data indicate that overactivated JNK signaling in skeletal muscle as present during the course of obesity fails to impair whole body glucose homeostasis and insulin-stimulated signaling events *in vivo*.

### Unaltered Energy Homeostasis and Body Composition in Mice Lacking JNK-1 in Skeletal Muscle

**Figure 4:**
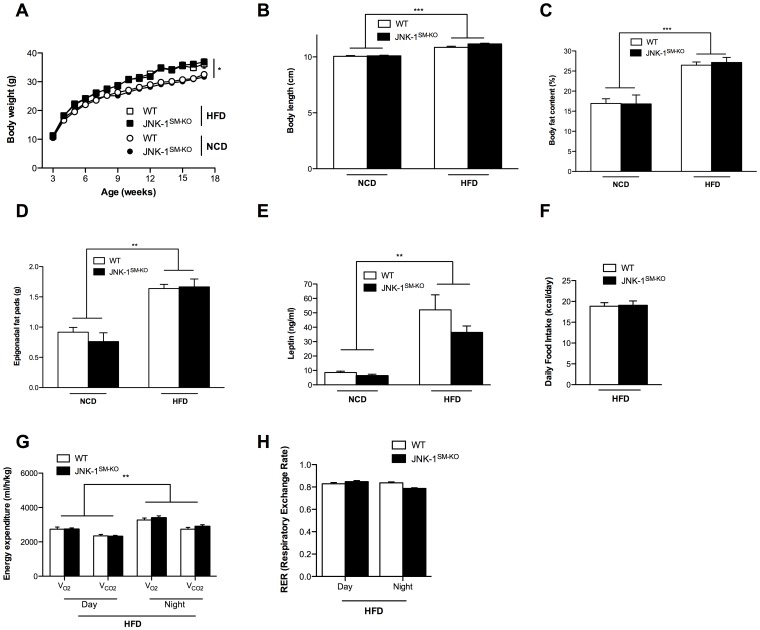
Unaltered body composition and energy homeostasis in JNK-1^SM-KO^ mice under normal and obese conditions. (A) The average bodyweight of control NCD fed (open circles) and HFD fed (open squares) mice was compared with JNK-1^SM-KO^ mice fed a NCD (black circles) or a HFD (black squares) from 3 to 17 weeks of age (n = 15–45). (B) Body length of control (white bar) and JNK-1^SM-KO^ (black bar) mice upon NCD and HFD feeding. (C) Body composition of control (white bar) and JNK-1^SM-KO^ (black bar) mice when exposed to NCD or HFD were determined by using a Brucker minispec in week 17 (n = 11–24). (D) Weight of epigonadal fat pads from NCD and HFD fed control (white bar) and JNK-1^SM-KO^ (black bar) mice in week 17 (n = 13–40). (E) Serum leptin levels of control (white bar) and JNK-1^SM-KO^ (black bar) mice upon NCD and HFD feeding at the age of 17 weeks (n = 8–18). (F) Daily food intake of control (white bar) and JNK-1^SM-KO^ (black bar) mice upon HFD feeding at the age of 14 weeks (n = 5–27). (G) Energy expenditure revealed by the daily and nightly volume of O_2_ consumption and CO_2_ release of control (white bar) and JNK-1^SM-KO^ (black bar) mice upon HFD feeding (n = 8). (H) Respiratory exchange rate (RER) of control (white bar) and JNK-1^SM-KO^ (black bar) mice upon HFD feeding (n = 8). Values are means ± SEM. **, p≤0.01; ***, p≤0.001.

The previously published analysis of conventional JNK-1 knock out mice had revealed, that these animals are not only protected from obesity-associated insulin resistance, but that they also exhibit reduced weight gain, both after exposure to high fat diet (HFD) as well as on the background of the *ob/ob* genetic obesity model [12]. To directly address whether skeletal muscle specific disruption of JNK-1 contributes to this effect, we investigated our recently described mice with skeletal muscle specific disruption of JNK-1 (JNK-1^SM-KO^ mice) [26]. To this end, we exposed cohorts of control and JNK-1^SM-KO^ mice to normal chow (NCD) and high fat diet (HFD) conditions starting from 3 weeks of age and monitored parameters of adiposity as well as energy homeostasis in these animals. Though this analysis revealed that HFD-exposure significantly increased body weight, body length, body fat content, epididymal fat pad mass as well as circulating plasma leptin concentrations in control mice exposed to HFD compared to those exposed to NCD (Fig. 4A, B, C, D, E), parameters of mice with skeletal muscle specific JNK-1 deficiency were indistinguishable from control mice, both under NCD and HFD conditions. Accordingly, while unaltered food intake and energy expenditure between control and JNK-1^SM-KO^ mice was demonstrated previously [26], also food intake and energy expenditure as well as respective respiratory exchange ratios were indistinguishable between JNK-1^SM-KO^ and control mice under obese conditions (Fig. 4F, G, H). In summary, our results clearly indicate that skeletal muscle-specific disruption of JNK-1 fails to affect body composition and energy homeostasis in lean and obese mice indicating that JNK-1-dependent signaling in other organs than skeletal muscle accounts for the protective effect of JNK-1 deficiency against the development of diet-induced obesity.

### Lack of JNK-1 in Skeletal Muscle does not Affect Glucose Homeostasis and Insulin Sensitivity Under Normal and Obese Conditions

**Figure 5:**
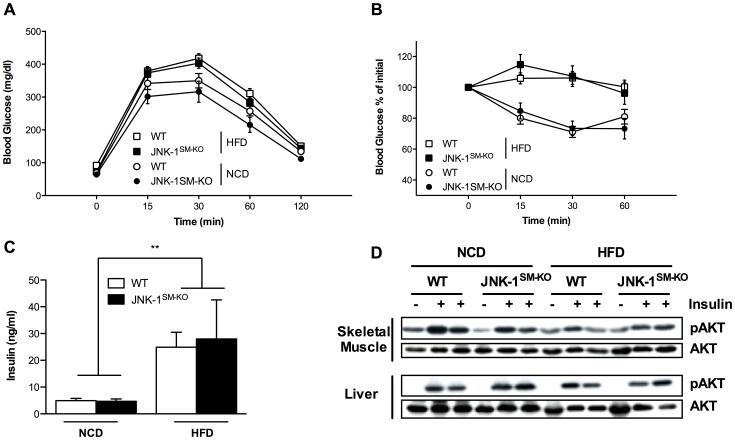
Unaltered glucose metabolism and insulin sensitivity in JNK-1^SM-KO^ under normal and obese conditions. (A) Glucose tolerance tests of control NCD fed (open circles) and HFD fed (open squares) mice and JNK-1^SM-KO^ mice fed a NCD (black circles) or a HFD (black squares) were performed at 11 weeks of age (n = 9–49). (B) Insulin tolerance tests of control NCD fed (open circles) and HFD fed (open squares) mice and JNK-1^SM-KO^ fed a NCD (black circles) or a HFD (black squares) were performed at 12 weeks of age (n = 5–30). (C) Insulin levels from sera isolated at week 17 from mice with the indicated genotypes upon NCD or HFD feeding determined by ELISA (n = 5–9). (D) Representative insulin-induced AKT phosphorylation of muscle and liver lysates isolated from control and JNK-1^SM-KO^ mice when feeding NCD and HFD using Western Blot analysis with the indicated antibodies. Values are means ± SEM. **, p≤0.01; ***, p≤0.001.

While JNK-1-deficiency in skeletal muscle did not affect the development of HFD-induced obesity, we next aimed to analyze, whether the absence of JNK-1-signaling in this organ affects the manifestation of obesity-associated insulin resistance. Exposure to HFD led to significantly impaired glucose and insulin tolerance in control mice compared to the NCD fed control cohort (Fig. 5A, B). However, when compared to the control groups of mice, glucose and insulin tolerance was unaltered in JNK-1^SM-KO^ mice both under lean and obese conditions (Fig. 5A, B). Moreover, while plasma insulin concentrations were significantly increased in obese control mice compared to NCD from 5 ng/ml to 25 ng/ml, identical results were obtained from mice with muscle specific JNK-1 deficiency (Fig. 5C). Thus, JNK-1-deficiency in skeletal muscle does not result in significant improvements of diet-induced deterioration of glucose metabolism in mice.

In the absence of alterations of whole body glucose metabolism in JNK-1^SM-KO^ mice compared to control mice, we next wanted to address, whether lack of JNK-1 in these organs may still lead to improved insulin signal transduction at a molecular level, which possibly fails to translate to changes in overall glucose metabolism. Thus, we assessed insulin’s ability to stimulate phosphorylation of AKT, as a measure for insulin-stimulated AKT activation *in vivo*. This analysis revealed impaired insulin-stimulated AKT-phosphorylation both in skeletal muscle and liver (Fig. 5D) of control mice exposed to HFD compared to lean controls exposed to NCD. However, the lack of JNK-1 in skeletal muscle could not improve the diet-induced inhibition of insulin-stimulated AKT-phosphorylation in these organs (Fig. 5D). Thus, our experiments unequivocally demonstrate, that JNK-1 is dispensable for the development of diet-induced insulin resistance in skeletal muscle of mice.

Noteworthy, our data are not in line with a previous report that identified a role of skeletal muscle specific JNK-1 in the development of diet-induced local and systemic insulin resistance (17). To assess whether this discrepancy might be a consequence of an alternative physiological characterization, we investigated control C57/BL6 mice in the different phenotyping protocols. To this end, cohorts of C57/BL6 mice were exposed to NCD and HFD starting with 3 weeks of age (WTS3), and compared to cohorts of C57/BL6 mice receiving the different diets with 8 weeks of age (WTS8), respectively. This analysis demonstrated that HFD exposure increased bodyweight gain, body length as well as adiposity in both protocols to a similar extent when compared to the NCD exposed control mice (Fig. S2A, B, C, D). The similar adiposity of WTS3 and WTS8 control mice exposed to HFD feeding could also be confirmed by the presence of similar circulating leptin levels as revealed by ELISA (Fig. S2E). Furthermore, while indirect calorimetry experiments revealed that WTS3 mice showed increased VO_2_ consumption and VCO_2_ production compared to the WTS8 counterparts mainly during the day phase under both dietary conditions, the respiratory exchange rate resulting from these data was indistinguishable (Fig. S2F, G). Nevertheless, diet-induced impairments of glucose homeostasis were similar in both protocols as revealed by glucose tolerance and insulin tolerance tests as well as increased circulating insulin concentrations (Fig. S2H, I, J, K, L). Collectively, these data demonstrate that the discrepancy between the previously published work (17) and our study is not a result of different physiological characterization protocols.

### Bodywide JNK-1 Deficiency Protects against HFD-induced Glucose Intolerance and Insulin Resistance

**Figure 6:**
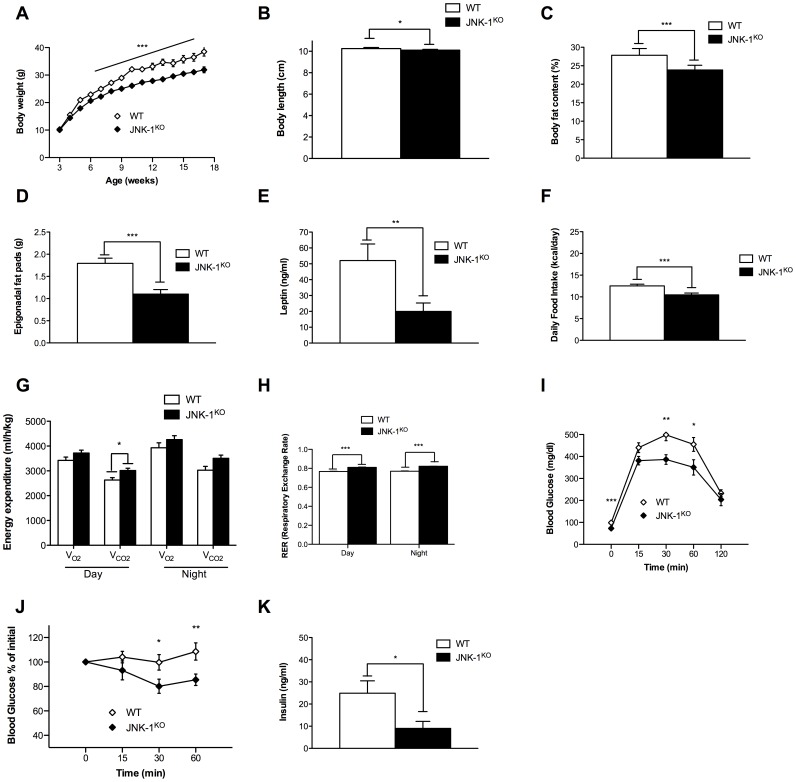
Complete absence of JNK-1 protects from HFD-induced obesity and insulin resistance. (A) The average body weight of WT (white diamonds) and JNK-1^KO^ (black diamonds) mice fed a HFD from 3 to 17 weeks of age (n = 12–18). (B) Body length of WT and JNK-1^KO^ mice upon HFD at 17 weeks age (n = 15). (C) Body composition of WT (white bar) and JNK-1^KO^ (black bar) mice when exposed to HFD was determined by using a Brucker minispec in week 17 (n = 15–19). (D) Weight of epigonadal fat pads from HFD fed WT (white bar) and JNK-1^KO^ (black bar) mice in week 17 (n = 10–12). (E) Serum leptin levels of WT (white bar) and JNK-1^KO^ (black bar) mice at the age of 17 weeks (n = 10). (F) Daily food intake of WT (white bar) and JNK-1^KO^ (black bar) mice upon HFD feeding at the age of 14 weeks (n = 17). (G) Energy expenditure revealed by the daily and nightly volume of O_2_ consumption and CO_2_ release of WT (white bar) and JNK-1^KO^ (black bar) mice upon HFD feeding (n = 5). (H) Respiratory exchange rate (RER) of control (white bar) and JNK-1^KO^ (black bar) mice upon HFD feeding (n = 5). (I) Glucose tolerance tests of WT (white diamonds) and JNK-1^KO^ (black diamonds) mice when feeding a HFD were performed at 11 weeks of age (n = 14). (J) Insulin tolerance tests of WT (white diamonds) and JNK-1^KO^ (black diamonds) mice upon HFD feeding were performed at 12 weeks of age (n = 14) (K) Insulin levels from sera isolated at week 17 from mice with the indicated genotypes upon HFD feeding determined by ELISA (n = 8). Values are means ± SEM, *, p<0.05, **, p<0.01, ***, p<0.001.

Disruption of JNK-1 by conventional means results in decreased obesity and improved glucose metabolism [12]. To confirm this important finding with our JNK-1 allele, we inactivated JNK-1 in the whole body by crossing JNK-1^FL/FL^ mice to deleter Cre mice (JNK-1^KO^ mice). Consistent with previously published results, JNK-1^KO^ mice on a NCD show not only unaltered bodyweight but also similar naso-anal lengths at 17 weeks of age when compared to controls (Fig. S3A, B). In line with these data, adiposity and epigonadal fat pad weights as well as circulating leptin levels and food ingestion were comparable between the two groups of mice (Fig. S3C, D, E, F). Moreover, while the parameters of energy homeostasis matched between control and JNK-1^KO^ mice (Fig. S3G, H), glucose tolerance and insulin sensitivity were largely unaffected by complete JNK-1 deficiency under normal conditions (Fig. S3I, J, K). As the beneficial effects of JNK-1 deficiency were observed under obese conditions, we assessed energy and glucose homeostasis in JNK-1^KO^ and control mice also when exposed to HFD feeding. Indeed, determination of body weight from weaning until 17 weeks of age of WT and JNK-1^KO^ mice revealed that JNK-1^KO^ mice are protected from HFD-induced weight gain (Fig. 6A). Moreover, since mice lacking JNK-1 in the CNS show a reduction in somatic growth as a consequence of impaired growth hormone/insulin like growth factor 1 axis [19], body length of WT and JNK-1^KO^ mice was assessed, revealing a significant reduction of body length in JNK-1^KO^ mice fed a HFD (Fig. 6B). The degree of adiposity in WT and JNK-1^KO^ mice upon HFD feeding was determined by NMR analysis and by measuring the weight of the epigonadal fat pads. These analyses demonstrated that JNK-1^KO^ mice exhibit decreased body fat content when compared to WT mice as well as reduced epigonadal fat pad weight (Fig. 6 C and D). Consistent with these findings, serum levels of leptin as an indirect measure of adiposity were reduced in JNK-1^KO^ mice when compared to WT mice (Fig. 6 E). Collectively, these results demonstrate that mice lacking JNK-1 in the whole body are protected from the development of HFD-induced adiposity. Reduced adiposity develops either in light of a reduced caloric intake or due to increases in energy expenditure or both. Noteworthy, the decreased adiposity of JNK-1^KO^ mice is a consequence of significantly reduced food intake of JNK-1^KO^ mice compared to controls (Fig. 6 F) rather than increased energy expenditure, whose means were only minor affected (Fig. 6 G). However, JNK-1^KO^ mice showed reduced HFD-induced lipid utilization as the daily and nightly RER was increased compared to controls (Fig. 6 H). Next, we wanted to address whether the reduction in adiposity in JNK-1^KO^ mice under obese conditions translates into improved glucose homeostasis. Hence, glucose and insulin tolerance tests were performed. This analysis revealed that the time-dependent glucose clearance from the blood upon an intraperitoneal glucose challenge was markedly ameliorated in JNK-1^KO^ mice when compared to control mice fed a HFD (Fig. 6 I). Moreover, insulin sensitivity as assessed by an insulin tolerance was strongly improved in JNK-1^KO^ mice when compared to WT mice (Fig. 6 J). Strikingly, JNK-1^KO^ mice were also protected from HFD-induced hyperinsulinemia as indicated by reduced circulating levels of insulin in comparison to WT mice (Fig. 6 K). In summary, these results clearly support previous work indicating that bodywide JNK-1 deficiency protects from diet-induced weight gain and insulin resistance. Thus, our experiments assign JNK-1 activation in tissues other than skeletal muscle a critical role in the development of obesity-associated deterioration of energy and glucose homeostasis.

## Discussion

The notion that obesity is associated with an increased inflammatory state, which activates numerous intracellular signaling cascades in turn inhibiting insulin action provides a unique opportunity for the development of novel therapeutic interventions to treat obesity-associated insulin resistance and diabetes. However, these novel therapeutic approaches require the exact definition of the intracellular signaling cascade activated by cytokines such as TNF-α and IL-6, ER-stress or altered lipid composition contributing to the inhibition of insulin signaling in a tissue-specific manner. Here, numerous candidate pathways activated by these stimuli have been shown to cause inhibitory serine phosphorylation of insulin receptor substrate proteins or to induce insulin resistance by other mechanisms [3], [4], [7], [8].

Thus, obesity-induced inflammatory signaling pathways include activation of IκBα kinases (IKK), the c-Jun N-terminal kinases (JNK), atypical protein kinase C (PKC) and it has been recently recognized that altered diacylglycerol (DAG) content in skeletal muscle can induce insulin resistance via diacylglycerol kinase (DGK) activation [27].

We and others could demonstrate that in liver, IKK activation plays an important function in the development of obesity-associated insulin resistance and that both, liver-specific IKK-2 or NEMO deficiency can protect from obesity-associated impairment of glucose tolerance [24], [28]. However, deletion of IKK-2 in skeletal muscle fails to prevent the development of obesity-associated insulin resistance, underlining the tissue-specific contribution of inflammatory mediators to cause insulin resistance [29].

In light of the dramatic protection from the occurrence of obesity-associated insulin resistance in conventional JNK-1-, but not JNK-2-deficient animals, JNK-1 was predicted to play an important role - particularly in the insulin-sensitive target tissue skeletal muscle - to cause obesity-associated insulin resistance [12]. Here we demonstrate what was also reported by others that obesity increases JNK activation in skeletal muscle that could potentially impair insulin signaling accounting for the development of obesity-associated insulin resistance in this tissue [17]. To elucidate this possibility, we successfully generated a mouse strain allowing for Cre-activated expression of a JNK constitutively active construct that we crossed to Mck-Cre mice to obtain skeletal muscle specific overactivation of JNK signaling as it is present under obese conditions. However, our metabolic data using these mice eliminate the assumption that increased JNK activation under obese conditions in skeletal muscle impairs insulin signaling but instead clearly communicate the important observation that obesity-induced JNK activation fails to impair insulin action in this organ. Despite similar skeletal muscle specific JNK overactivation in these mice as it is present under obese conditions, these mice lacked any alteration in adiposity, glucose metabolism and energy homeostasis. In line with this observation, we also characterized mice with isolated JNK-1 deficiency in skeletal muscle under lean and obese conditions in which none of the assessed metabolic parameters were affected. Thus, our current finding that isolated JNK overactivation and JNK-1 deficiency in skeletal muscle fails to protect from obesity-associated disturbances in overall glucose metabolism as assessed during glucose tolerance and insulin tolerance tests is surprising. Several explanations may account for this phenomenon. An obvious possibility would be redundant signaling functions of other JNK isoforms in the absence of JNK-1. Therefore, in skeletal muscle, JNK-2 activation may compensate for the lack of JNK-1 and thereby cause development of insulin resistance upon obesity-induction. Indeed, it could be demonstrated that compound deficiency for JNK-1 and heterozygousity for a conventional JNK-2 null allele has a more profound effect to protect from diet-induced insulin resistance than isolated JNK-1 deficiency [13]. Thus, in skeletal muscle either JNK-1 and JNK-2 contribute cooperatively to the development of obesity-associated insulin resistance or alternative kinases such as atypical PKC- and DGK-activation may play a more important role in the development of obesity-induced insulin resistance in this organ [27], [30]. An alternative explanation that in our settings JNK-1 deficiency in skeletal muscle fails to improve obesity-associated disorders might be the diverging gene targeting strategies used by us and others [17], [19]. Though the previously described JNK-1 conventional knock out mouse replaces exon 2 of JNK-1 with a neo resistance gene [12] and we copied this strategy by flanking exon 2 of JNK-1 with loxP sites, we assessed whether the absence of JNK-1 in the whole body indeed protects from diet-induced obesity and associated disorders. Consistent with the report of Hirosoumi and colleagues [12], also our JNK-1 deficient mouse model showed protection against the development of diet-induced weight gain and showed improved insulin sensitivity.

Thus, what remains a striking observation requiring further investigation is the profound difference of the metabolic phenotype of conventional JNK-1-deficient mice and the reported phenotypes for mice with cell type-specific disruption of JNK-1 activity. While our report clearly demonstrates that skeletal muscle-specific JNK-1 deficiency is not sufficient to protect against obesity-associated insulin resistance, a recent report indicates that inactivation of JNK-1 in the adipose tissue causes some degree of protection from obesity-associated insulin resistance due to the inactivation of JNK-1 in adipose tissue [7]. In the latter study, adipocyte-autonomous JNK-1 signaling regulates IL-6 secretion in the obese white adipose tissue that in turn inhibits hepatic insulin action through IL-6-induced SOCS-3 expression in the liver [31], [32]. Noteworthy, we demonstrated recently that JNK-1 signaling in skeletal muscle controls IL-6 expression in response to exercise [26] implicating an important function of JNK-1-induced IL-6 expression also in other cell types. However, JNK-1-deficient myeloid lineage cells show unaltered IL-6 expression and JNK-1 deficiency in these cells *per se* had no effect on the development of obesity-associated insulin resistance whereas other reports on bone marrow transplantation chimeras with JNK-1-deficient hematopoetic stem cells have yielded controversial findings [14]–[16]. Nevertheless, neither adipocyte-specific disruption nor hematopoetic-specific JNK-1-deficiency phenocopies the effect of conventional JNK-1 deletion. In line with these reports, also JNK-1 deficiency in liver parenchymal cells has failed to affect body weight gain and insulin sensitivity, but demonstrated an important function of JNK-1 in the prevention of hepatic lipid accumulation [18]. Another candidate organ where the absence of JNK-1 could protect from diet-induced obesity is the central nervous system (CNS). The CNS plays a central role in the control of body weight and peripheral glucose metabolism [33], [34]. Within the CNS, insulin action particularly in agouti-related peptide-expressing neurons of the arcuate nucleus of the hypothalamus controls peripheral glucose metabolism via inhibition of hepatic glucose production [35]. Thus, JNK-mediated inhibition of insulin signal transduction in the CNS may play an important role in deterioration of peripheral glucose metabolism. Essentially, we have demonstrated that the combined inactivation of JNK-1 in the CNS and the pituitary represents as yet the most promising approach to resemble the phenotype of conventional JNK-1 knock out mice [19]. Though in these mice adiposity is unaffected in response to HFD, systemic insulin sensitivity is improved accompanied with reduced somatic growth. Taken together, our experiments provide evidence for an important role of JNK-1 signaling in organs other than skeletal muscle in the development of obesity-associated insulin resistance and diabetes.

## Supporting Information

Figure S1 **Transgenic JNK^C^ expression in skeletal muscle enhances phosphorylation of cJun.** (A) JNK-kinase assay (KA) of protein lysates isolated from muscles of WT and JNK^SM-C^ mice fed a NCD. Calnexin antibodies were used for input control of the lysates. (B) Quantitation of radioactively labeled cJun peptide in skeletal muscle of NCD fed WT and JNK^SM-C^ mice. The data were adjusted to the WT NCD data shown in figure 1 B, C. Values are means ± SEM. *, p≤0.05.
(TIF)

Figure S2 **Physiological comparison of diet-induced obesity protocols.** (A) The average bodyweight of WTS3 NCD fed (open squares) and WTS3 HFD fed (grey circles) mice was compared with WTS8 mice fed a NCD (open squares) or a HFD (grey circles) from 0 to 17 weeks on both diets, respectively (n = 10). (B) Body length of WTS3 (white bar) and WTS8 (grey bar) mice upon NCD and HFD feeding (n = 10). (C) Body composition of WTS3 (white bar) and WTS8 (grey bar) mice when exposed to NCD or HFD was determined by using a Brucker minispec in week 17 of feeding the diets (n = 10). (D) Weight of epigonadal fat pads from NCD and HFD WTS3 (white bar) and WTS8 (grey bar) mice in week 17 of feeding the diets (n = 10). (E) Serum leptin levels of WTS3 (white bar) and WTS8 (grey bar) mice upon NCD and HFD feeding after 17 weeks on both the diets (n = 10). (F) Energy expenditure revealed by the daily and nightly volume of O_2_ consumption and CO_2_ release of WTS3 (white bar) and WTS8 (grey bar) mice upon NCD and HFD feeding (n = 10). (G) Respiratory exchange rate (RER) of WTS3 (white bar) and WTS8 (grey bar) mice upon NCD and HFD feeding (n = 10). (H) Glucose tolerance tests of WTS3 NCD fed (open squares) and HFD fed (open circles) mice were performed after 17 weeks on either diet (n = 10). (I) Glucose tolerance tests of WTS8 NCD fed (grey squares) and HFD fed (grey circles) mice were performed after 17 weeks on either diet (n = 10). (J) Insulin tolerance tests of WTS3 NCD fed (open squares) and HFD fed (open circles) mice were performed after 17 weeks on either diet (n = 10). (K) Insulin tolerance tests of WTS8 fed a NCD (grey squares) or a HFD (grey circles) were performed after 17 weeks on either diet (n = 10). (L) Insulin levels from sera isolated after 17 weeks on either of the diets from mice with the indicated genotypes determined by ELISA (n = 10). Values are means ± SEM. **, p≤0.01; ***, p≤0.001.
(TIF)

Figure S3 **Phenotypical analysis of bodywide JNK-1 deficiency under normal conditions.** (A) The average body weight of WT (white bars) and JNK-1^KO^ (black bars) mice fed a NCD at 17 weeks of age (n = 5). (B) Body length of WT and JNK-1^KO^ mice upon NCD feeding at 17 weeks age (n = 5). (C) Body composition of WT (white bar) and JNK-1^KO^ (black bar) mice when exposed to NCD was determined by using a Brucker minispec in week 17 (n = 5). (D) Weight of epigonadal fat pads from NCD fed WT (white bar) and JNK-1^KO^ (black bar) mice in week 17 (n = 5). (E) Serum leptin levels of WT (white bar) and JNK-1^KO^ (black bar) mice at the age of 17 weeks (n = 5). (F) Daily food intake of WT (white bar) and JNK-1^KO^ (black bar) mice upon NCD feeding at the age of 14 weeks (n = 5). (G) Energy expenditure revealed by the daily and nightly volume of O_2_ consumption and CO_2_ release of WT (white bar) and JNK-1^KO^ (black bar) mice upon NCD feeding (n = 5). (H) Respiratory exchange rate (RER) of control (white bar) and JNK-1^KO^(grey bar) mice upon NCD feeding (n = 5). (I) Glucose tolerance tests of WT (white diamonds) and JNK-1^KO^ (black diamonds) mice when feeding a NCD were performed at 11 weeks of age (n = 5). (J) Insulin tolerance tests of WT (white diamonds) and JNK-1^KO^ (black diamonds) mice upon NCD feeding were performed at 12 weeks of age (n = 5) (K) Insulin levels from sera isolated at week 17 from mice with the indicated genotypes upon NCD feeding determined by ELISA (n = 5). Values are means ± SEM, *, p<0.05.
(TIF)
