# Supplementary figures and images for: Alteration of JNK-1 Signaling in Skeletal Muscle Fails to Affect Glucose Homeostasis and Obesity-Associated Insulin Resistance in Mice
Source: PLoS One. 2013 Jan 17;8(1):e54247. doi: 10.1371/journal.pone.0054247 (PMC3547909; doi:10.1371/journal.pone.0054247)

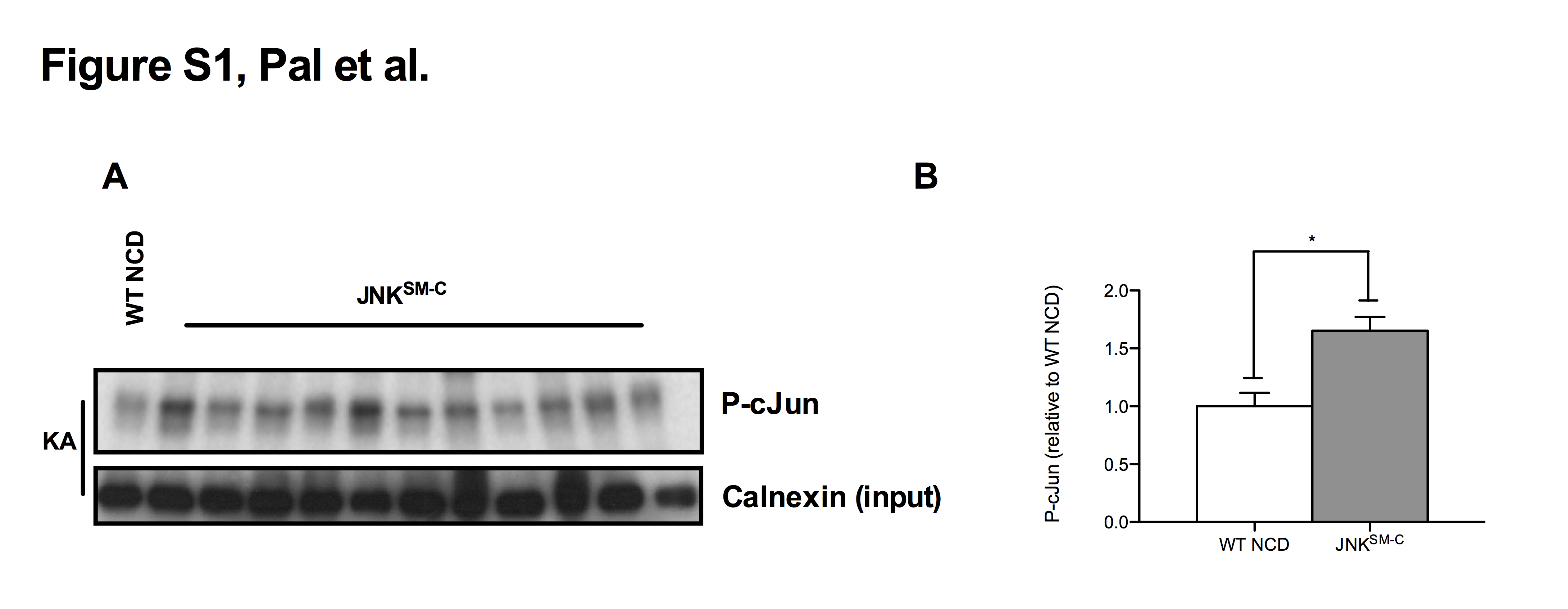

Supplement: Figure S1 — Transgenic JNKC expression in skeletal muscle enhances phosphorylation of cJun. (A) JNK-kinase assay (KA) of protein lysates isolated from muscles of WT and JNKSM-C mice fed a NCD. Calnexin antibodies were used for input control of the lysates. (B) Quantitation of radioactively labeled cJun peptide in skeletal muscle of NCD fed WT and JNKSM-C mice. The data were adjusted to the WT NCD data shown in figure 1 B, C. Values are means ± SEM. *, p≤0.05. (TIF) [file pone.0054247.s001.tif]

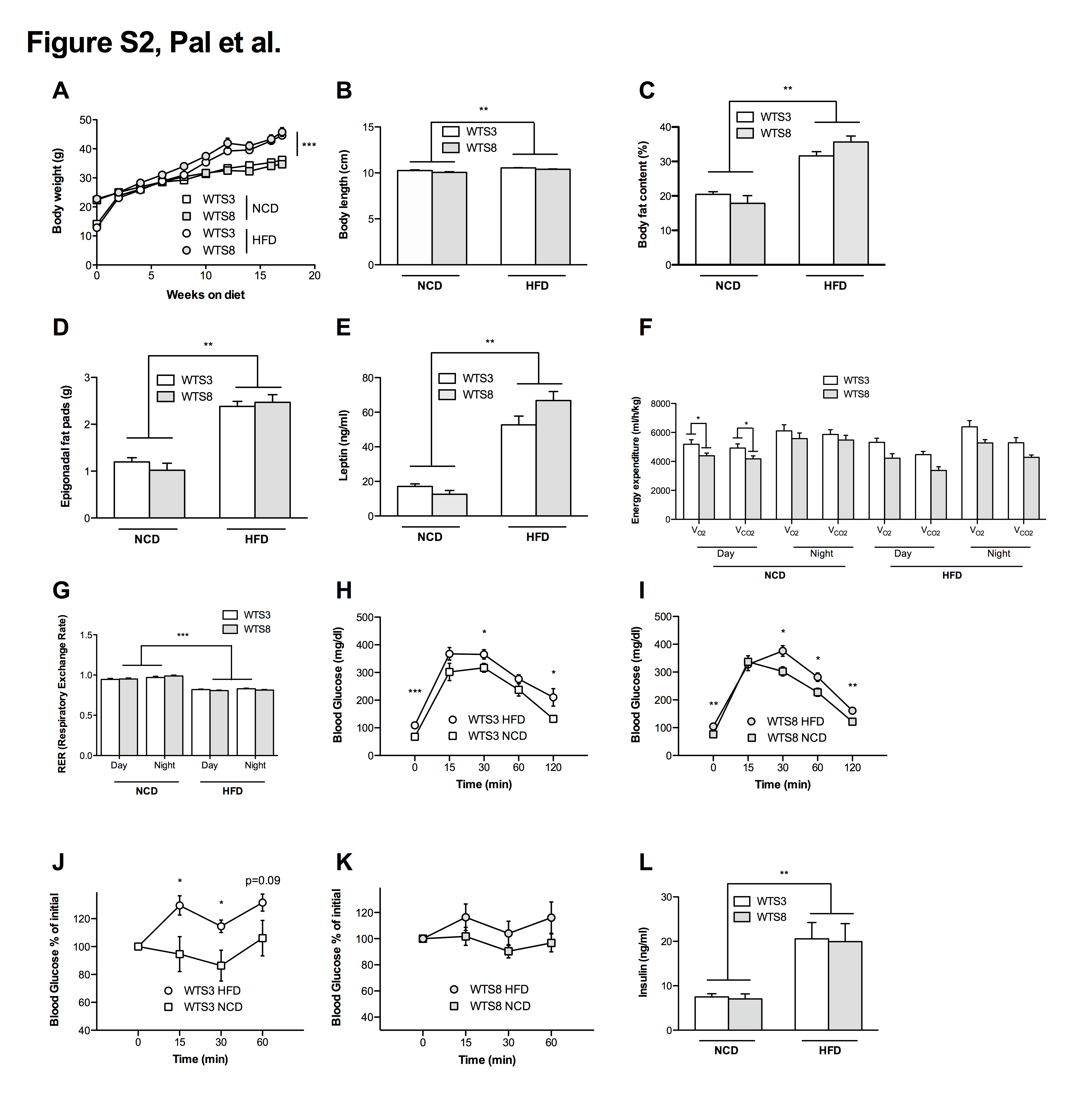

Supplement: Figure S2 — Physiological comparison of diet-induced obesity protocols. (A) The average bodyweight of WTS3 NCD fed (open squares) and WTS3 HFD fed (grey circles) mice was compared with WTS8 mice fed a NCD (open squares) or a HFD (grey circles) from 0 to 17 weeks on both diets, respectively (n = 10). (B) Body length of WTS3 (white bar) and WTS8 (grey bar) mice upon NCD and HFD feeding (n = 10). (C) Body composition of WTS3 (white bar) and WTS8 (grey bar) mice when exposed to NCD or HFD was determined by using a Brucker minispec in week 17 of feeding the diets (n = 10). (D) Weight of epigonadal fat pads from NCD and HFD WTS3 (white bar) and WTS8 (grey bar) mice in week 17 of feeding the diets (n = 10). (E) Serum leptin levels of WTS3 (white bar) and WTS8 (grey bar) mice upon NCD and HFD feeding after 17 weeks on both the diets (n = 10). (F) Energy expenditure revealed by the daily and nightly volume of O2 consumption and CO2 release of WTS3 (white bar) and WTS8 (grey bar) mice upon NCD and HFD feeding (n = 10). (G) Respiratory exchange rate (RER) of WTS3 (white bar) and WTS8 (grey bar) mice upon NCD and HFD feeding (n = 10). (H) Glucose tolerance tests of WTS3 NCD fed (open squares) and HFD fed (open circles) mice were performed after 17 weeks on either diet (n = 10). (I) Glucose tolerance tests of WTS8 NCD fed (grey squares) and HFD fed (grey circles) mice were performed after 17 weeks on either diet (n = 10). (J) Insulin tolerance tests of WTS3 NCD fed (open squares) and HFD fed (open circles) mice were performed after 17 weeks on either diet (n = 10). (K) Insulin tolerance tests of WTS8 fed a NCD (grey squares) or a HFD (grey circles) were performed after 17 weeks on either diet (n = 10). (L) Insulin levels from sera isolated after 17 weeks on either of the diets from mice with the indicated genotypes determined by ELISA (n = 10). Values are means ± SEM. **, p≤0.01; ***, p≤0.001. (TIF) [file pone.0054247.s002.tif]

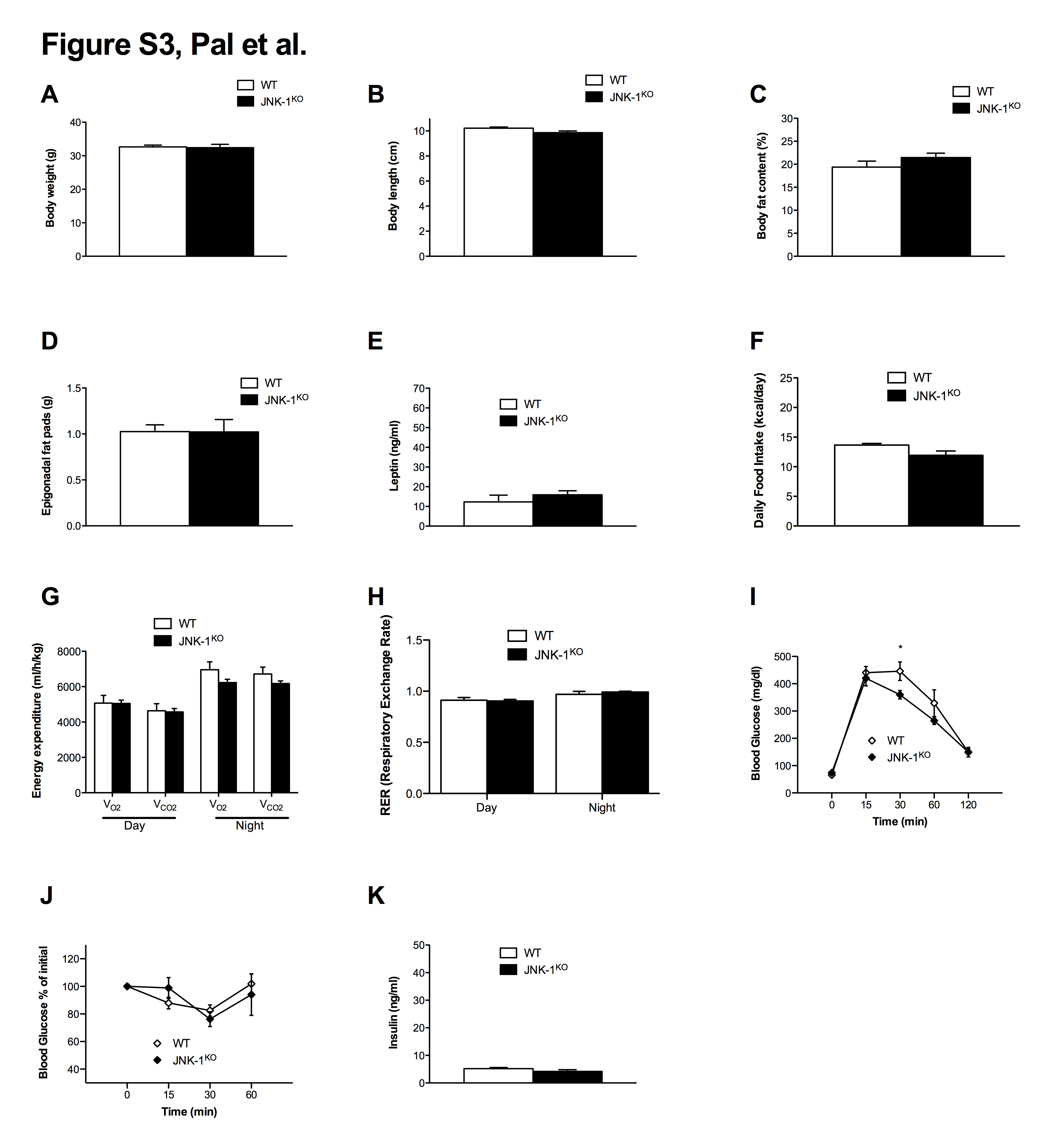

Supplement: Figure S3 — Phenotypical analysis of bodywide JNK-1 deficiency under normal conditions. (A) The average body weight of WT (white bars) and JNK-1KO (black bars) mice fed a NCD at 17 weeks of age (n = 5). (B) Body length of WT and JNK-1KO mice upon NCD feeding at 17 weeks age (n = 5). (C) Body composition of WT (white bar) and JNK-1KO (black bar) mice when exposed to NCD was determined by using a Brucker minispec in week 17 (n = 5). (D) Weight of epigonadal fat pads from NCD fed WT (white bar) and JNK-1KO (black bar) mice in week 17 (n = 5). (E) Serum leptin levels of WT (white bar) and JNK-1KO (black bar) mice at the age of 17 weeks (n = 5). (F) Daily food intake of WT (white bar) and JNK-1KO (black bar) mice upon NCD feeding at the age of 14 weeks (n = 5). (G) Energy expenditure revealed by the daily and nightly volume of O2 consumption and CO2 release of WT (white bar) and JNK-1KO (black bar) mice upon NCD feeding (n = 5). (H) Respiratory exchange rate (RER) of control (white bar) and JNK-1KO(grey bar) mice upon NCD feeding (n = 5). (I) Glucose tolerance tests of WT (white diamonds) and JNK-1KO (black diamonds) mice when feeding a NCD were performed at 11 weeks of age (n = 5). (J) Insulin tolerance tests of WT (white diamonds) and JNK-1KO (black diamonds) mice upon NCD feeding were performed at 12 weeks of age (n = 5) (K) Insulin levels from sera isolated at week 17 from mice with the indicated genotypes upon NCD feeding determined by ELISA (n = 5). Values are means ± SEM, *, p<0.05. (TIF) [file pone.0054247.s003.tif]
